# Liver Transplantation for Unresectable Biliary Tract Cancers: Largest Single-Center Analysis of Subtype-Specific Survival and Recurrence (2007–2025)

**DOI:** 10.1245/s10434-026-19975-6

**Published:** 2026-06-20

**Authors:** Sally Campbell, Ola Ahmed, Darren Cullinan, Neeta Vachharajani, Adeel S. Khan, William C. Chapman, Majella B. M. Doyle

**Affiliations:** Department of Abdominal Organ Transplantation Surgery, Washington University School of Medicine, St Louis, MO USA

## Abstract

**Background:**

In carefully selected patients, liver transplantation (LT) has provided encouraging outcomes for unresectable biliary tract cancers (BTC), including biphenotypic (cHCC-CC), perihilar (pCC), and intrahepatic cholangiocarcinoma (iCCA). This study aimed to characterize diagnostic discrepancies, recurrence patterns, and oncologic metrics in this unique LT population.

**Patients and Methods:**

Patients with BTC receiving LT within a single academic transplant center were included (*n* = 98 from 2007 to 2025). Survival and recurrence outcomes were analyzed by tumor histotype using Kaplan–Meier and log-rank tests. Univariate and multivariate Cox proportional hazards regression was performed.

**Results:**

The most common transplanted subtype was pCC (*n* = 54, 55.1%), followed by cHCC-CC (*n* = 28, 28.6%) and iCCA (*n* = 16, 16.3%). Pretransplant diagnoses frequently differed from final explant pathology in 26 patients (26.5%). Recurrence occurred in 40 (40.8%) patients with a median time to recurrence of 16 months (interquartile range 1–160 months). Estimated 1-, 3-, and 5-year overall survival rates for the entire cohort were 88%, 68%, and 58%, respectively. On multivariate analysis, lymph node positivity (hazard ratio [3.27, *p* = 0.010), iCCA subtype (HR 2.94, *p* = 0.030), and explant tumor size (HR 1.22 per cm, *p* = 0.005) were independent predictors of recurrence.

**Conclusions:**

This is, to our knowledge, the largest single-center analysis of liver transplant recipients with BTC. Lymph node positivity, iCCA subtype, and tumor size are independent predictors of recurrence following LT for BTC. Despite the diagnostic challenges and high disease recurrence, LT is a promising option for unresectable BTC when no other surgical option exists.

Biliary tract cancers (BTC) encompass a heterogeneous group of malignancies, including perihilar cholangiocarcinoma (pCC), intrahepatic cholangiocarcinoma (iCCA), and combined hepatocellular-cholangiocarcinoma (cHCC-CC). Historically, the prognosis of unresectable BTCs has been dismal owing to limited surgical options, aggressive tumor biology, and high recurrence rates.^1^ Liver transplantation (LT) has emerged as a curative strategy for selected patients with unresectable BTC, offering complete tumor excision, removal of occult intrahepatic disease, and restoration of liver function.^2^ Transplantation is particularly promising for patients with locally advanced or small tumors demonstrating a favorable response to multimodal treatment regimens, including neoadjuvant chemotherapy and radiotherapy.^3–5^ Despite encouraging survival in select cohorts, adverse tumor biology expressed by high recurrence rates continues to limit overall effectiveness of LT in BTC.^6^

Understanding the clinicopathologic patterns associated with post-transplant recurrence is crucial to refining selection criteria and developing evidence-based protocols that maximize long-term outcomes. While single-center experiences have increased insights into the interplay between tumor biology, recurrence, and survival following LT for BTCs, granular data on prognostic factors and subtype-specific recurrence patterns remain scarce. This retrospective study provides an 18-year single-center experience characterizing diagnostic discrepancies, recurrence patterns, and oncologic outcomes following LT for BTC.

## Patients and Methods

A prospectively maintained clinicopathologic database was reviewed for all patients undergoing LT with an explant diagnosis of BTC between January 2007 and October 2025. Criteria for unresectability were subtype-specific. For pCC, unresectability criteria include bilateral hepatic duct involvement (Bismuth-Corlette type IV), unilateral duct involvement with contralateral portal vein involvement, or main portal vein involvement. For iCCA, unresectability criteria include bilobar disease, tumor proximity to major vascular structures precluding R0 resection, or insufficient future liver remnant, including patients with cirrhosis precluding major hepatectomy.^7^ The majority of cHCC-CC cases (27/28) were diagnosed as HCC pretransplant and were transplanted under standard HCC indications. All patients with unresectable liver malignancies are discussed at a multidisciplinary meeting with hepatopancreatobiliary and transplant surgical expertise to determine surgical and nonsurgical options.

Overall, 98 patients were included for analysis. Cancer staging followed AJCC guidelines.^8^ Patients were grouped by explant tumor diagnosis. Primary outcomes were overall survival (OS) and disease recurrence; secondary outcomes included recurrence patterns, post-recurrence treatment, and mortality by subtype. Institutional Review Board (IRB) approval was obtained.

### Treatment and Postoperative Surveillance

Pretransplant imaging evaluation included contrast-enhanced computed tomography (CT) and/or magnetic resonance imaging (MRI) of the chest and abdomen. Imaging protocols evolved over the study period in accordance with advances in cross-sectional imaging technology, which may have improved characterization of hepatic lesions and biliary anatomy. Patients with confirmed pCC underwent preoperative staging with CT or MRI of the chest and abdomen, endoscopic ultrasound-guided lymph node sampling, and staging laparoscopy. Inclusion criteria for LT in pCC include a malignant-appearing stricture with at least one of the following: positive or highly suspicious biopsy/cytology including polysomy by FISH, CA 19-9 > 100 U/mL in the absence of acute bacterial cholangitis, a well-defined mass with radial diameter < 3 cm on cross-sectional imaging, or tumor-negative lymph nodes during staging laparoscopy.^4^ Patients with confirmed pCC then underwent standardized neoadjuvant chemoradiation. Treatment included gemcitabine-based chemoradiation with serial staging laparoscopy, and patients with evidence of extrahepatic spread were excluded.^4^ Patients with liver-only unresectable iCCA, regardless of size, received neoadjuvant treatment in the form of systemic chemotherapy combined with liver-directed therapy, hepatic artery infusion pump insertion, and/or external beam radiation. Transplant eligibility was determined by disease response per RECIST criteria and a minimum of 6 months of disease stability with no extrahepatic spread.^9^ Treatment response was monitored by restaging cross-sectional imaging (CT and/or MRI) at defined intervals during neoadjuvant therapy, with chest CT and liver MRI repeated every 3 months while candidates awaited transplantation, as previously described.^4^

Post-LT, patients received standard dual immunosuppression with an antimetabolite and tacrolimus, with mTOR inhibitor substitution when indicated. A short prednisolone taper was discontinued by 6 weeks. Patients were reviewed every 2 weeks for 3 months, then every 3 months for 1 year, and every 6 months up to 5 years, including chest CT, abdominal MRI, and CA 19-9 at each 6-month visit.^4^

### Statistical Analyses

Continuous variables were presented as means with standard deviations for normally distributed data and as medians with ranges for skewed distributions. Categorical variables were summarized as frequencies and percentages. Overall survival and recurrence estimates were calculated by Kaplan–Meier analysis and compared using the log-rank test. Univariate Cox proportional hazards regression was performed for candidate predictors including age, tumor subtype, T stage, lymph node positivity, explant tumor size, CA 19-9, MELD, histologic grade, waitlist time, cirrhosis, and neoadjuvant chemoradiation. Variables with *p* < 0.20 on univariate analysis, along with clinically relevant covariates, were included in multivariate models for OS and recurrence-free survival (RFS). The number of covariates was limited to maintain approximately 10 events per variable. A sensitivity analysis including pretransplant CA 19-9 was performed on the subset with available data (*n* = 70). Model discrimination was assessed using the concordance index. Tumor size was modeled as a continuous covariate; however, the biological significance of tumor size differs across BTC subtypes, with radial dimension carrying specific UNOS policy significance for pCC (≤ 3 cm), whereas volumetric tumor size has different implications for iCCA and cHCC-CC. A two-sided *p* < 0.05 was considered significant. Analyses were performed using IBM SPSS v25.0.

## Results

### Patient Demographics and Clinical Characteristics

**Fig. 1:**
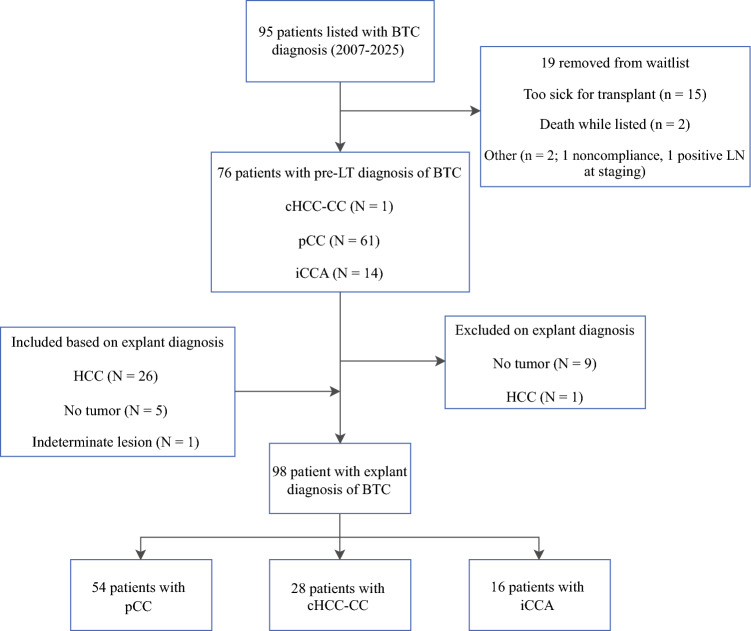
Flowchart of the study. *cHCC-CC* combined hepatocellular cholangiocarcinoma, *HCC* hepatocellular carcinoma, *pCC* perihilar cholangiocarcinoma, *iCCA* intrahepatic cholangiocarcinoma, *LT* liver transplant, *BTC* biliary tract cancer

Among 95 patients listed with a cholangiocarcinoma diagnosis at our center between 2007 and September 2025, 19 (20.0%) were removed from the waitlist prior to transplant. The majority of removals were pCC (*n* = 18, 94.7%) with one iCCA (5.3%). Tumor progression or metastatic disease was the most common reason for removal (*n* = 15, 78.9%), followed by medical decline or frailty (*n* = 3, 15.8%) and noncompliance (*n* = 1, 5.3%); three patients with pCC were taken to the operating room but had transplantation aborted intraoperatively owing to metastatic disease discovered at exploration (Fig. 1). Of the 76 transplanted, 10 were excluded for no BTC or reclassification on explant. An additional 32 patients with incidental BTC on explant were included, yielding a final cohort of 98 patients (mean age 60 ± 11 years): pCC (*n* = 54, 55.1%), cHCC-CC (*n* = 28, 28.6%), and iCCA (*n* = 16, 16.3%) (Fig. 1). Clinicopathologic characteristics are presented in Table 1. A total of 44 (44.9%) patients had no preexisting liver disease. HCV-related liver disease was the most common etiology (*n* = 18, 18.4%), followed by PSC (*n* = 13, 13.3%). The median calculated MELD at transplant was 11 [interquartile range (IQR) 6–47], and was highest for cHCC-CC at 18 (*p* = 0.014). The median BTC tumor size at explant was 2.3 cm [IQR 0–9 cm]. Overall, 81 patients (82.7%) had cirrhosis; all 28 patients with cHCC-CC had cirrhosis versus 39/54 of patients with pCC (72.2%, *p* = 0.009).

**Table 1:** Clinical and pathological characteristics based on explant tumor diagnosis

	cHCC-CC (*n* = 28)	pCC (*n* = 54)	iCCA (*n* = 16)	Total (*n* = 98)	*p* value
Age at transplant, mean ± SD (years)	62 ± 11	58 ± 11	63 ± 12	60 ± 11.2	0.227
Male, *n* (%)	23 (82.1)	41 (75.9)	10 (62.5)	74 (75.5)	0.344
Pre-existing liver disease, *n* (%)					
None	0	37 (68.5)	7 (43.8)	44 (45.8)	< 0.001
ETOH	6 (23.1)	0	4 (25)	10 (10.5)	< 0.001
NASH	5 (19.2)	2 (3.8)	2 (12.5)	9 (9.5)	0.079
HCV	15 (53.6)	1 (1.9)	2 (12.5)	18 (18.6)	< 0.001
PSC	0	12 (22.2)	1 (7.7)	13 (13.7)	0.014
Other^a^	4 (15.4)	1 (1.9)	2 (12.5)	7 (7.4)	0.067
Calculated MELD median [range]	18 [6–47]	9 [6–32]	12 [6–30]	11 [6–47]	0.014
UNOS exception MELD median [range]	26 [7–38]	24 [6–32]	16 [6–29]	25 [6–38]	< 0.001
Bilirubin mg/dL, mean ± SD	4.1 (5.2)	2.9 (5.5)	4.1 (9.1)	3.4 (6.1)	0.635
INR, mean ± SD	1.8 (1.4)	1.3 (0.3)	1.3 (0.4)	1.4 (0.8)	0.013
CA 19-9 (U/mL), median [range]	20.1 [3.6–846.5]	56.1 [1.9–989.4]	30.95 [8.7–1035]	47.5 [1.9–1035]	0.003
Tumor size (cm), median [range]					
At diagnosis	2.3 [0.9–7]	2.2 [0.7–4]	4.8 [1.2–12.2]	2.45 [0.7–12.2]	0.150
Post neoadjuvant therapy	2.3 [0–4.7]	2 [0.8–4.2]	2.4 [0–3.5]	2.2 [0–4.7]	0.492
At explant	2.5 [0.1–5.5]	2.2 [0–9]	1.2 [0–6.5]	2.3 [0–9]	0.503
Hepatitis status, *n* (%)					
Hepatitis C recipient (anti-HCV)	11 (39.3)	2 (3.7)	3 (18.8)	16 (16.3)	< 0.001
Hepatitis B recipient (anti-HBcAb)	4 (14.3)	1 (1.9)	2 (12.5)	7 (7.1)	0.077
Hepatitis C donor (anti-HCV)	10 (35.7)	4 (7.4)	3 (18.8)	17 (17.3)	0.006
Hepatitis B donor (anti-HBcAb)	7 (25)	2 (3.7)	0	9 (9.2)	0.003
Pretransplant therapy, *n* (%)					
Any pretransplant therapy	23 (82.1)	52 (96.3)	14 (87.5)	89 (90.8)	0.096
Institutional chemoRT protocol^b^	3 (10.7)	45 (83.3)	0	48 (49.0)	< 0.001
TACE	18 (64.3)	2 (3.7)	1 (6.2)	21 (21.4)	< 0.001
Y90 radioembolization	4 (14.3)	0	8 (50.0)	12 (12.2)	< 0.001
Ablation	4 (14.3)	0	1 (6.2)	5 (5.1)	0.020
SBRT	1 (3.6)	29 (53.7)	2 (12.5)	32 (32.7)	< 0.001
Systemic chemotherapy	2 (7.1)	5 (9.3)	3 (18.8)	10 (10.2)	0.446
HAIP	0	0	2 (12.5)	2 (2.0)	0.005
Previous resection	0	1 (1.9)	0	1 (1.0)	0.663
Waitlist time (days), median [range]	121 [2–1096]	66 [5–1290]	129 [5–463]	100 [2–1290]	0.022
Explant pathologic T stage, *n* (%)					
T0	0	6 (11.1)	0	6 (6.1)	0.074
T1	15 (53.6)	20 (37)	13 (81.3)	48 (49)	0.007
T2	11 (39.3)	19 (35.2)	2 (12.5)	32 (32.7)	0.159
T3	1 (3.6)	2 (3.7)	1 (6.3)	4 (4.1)	0.891
T4	1 (3.6)	7 (13)	0	8 (8.2)	0.144
Number of examined LNs, median [range]	1 [0-2]	4 [0–30]	0 [0–8]	2 [0–30]	< 0.001
LN positive, median [range]	0	0 [0–3]	0 [0–1]	0 [0–3]	0.150
Cirrhosis, *n* (%)	28 (100)	39 (73.6)	14 (87.5)	81 (83.5)	0.009
Path tumor grade, *n* (%)					
Well-differentiated	0	5 (10.2)	0	5 (5.9)	0.142
Moderately differentiated	13 (54.2)	23 (46.9)	6 (50)	42 (49.4)	0.844
Poorly differentiated	6 (25)	11 (22.4)	3 (25)	20 (23.5)	0.963
90 day mortality, *n* (%)	0	1 (1.9)	1 (6.3)	2 (2)	0.366
Time period of surgery, *n* (%)					
Before 2011	1 (3.6)	5 (9.3)	1 (6.3)	7 (7.1)	0.631
2011–2015	14 (50)	12 (22.2)	1 (6.3)	27 (27.6)	0.003
2016–2020	10 (35.7)	19 (35.2)	3 (18.8)	32 (32.7)	0.431
2021–2025	3 (10.7)	18 (33.3)	11 (68.8)	32 (32.7)	< 0.001

^a^Hepatitis B infection, Budd–Chiari syndrome, cryptogenic liver disease, alpha-1-anti-trypsin deficiency, secondary sclerosing liver disease, unspecified cholestatic liver disease

^b^Standardized gemcitabine-based neoadjuvant chemoradiation protocol for pCC (three patients with cHCC-CC initially diagnosed as hilar CCA also completed this protocol)

*cHCC-CC* combined hepatocellular cholangiocarcinoma, *HCC* hepatocellular carcinoma, *pCC* perihilar cholangiocarcinoma, *iCCA* intrahepatic cholangiocarcinoma, *SD* standard deviation, *UNOS* United Network for Organ Sharing, *HAIP* hepatic artery infusion pump, *LN* lymph node, *ETOH* alcohol related liver disease, *NASH* nonalcohol related steatohepatitis, *HCV* hepatitis C related liver disease, *PSC* primary sclerosing cholangitis, *MELD* Model for End-Stage Liver Disease

Overall, 42 tumors (49.4%) were moderately differentiated at explant and 20 (23.5%) were poorly differentiated, with no significant differences in tumor grade between BTC groups. iCCA had the highest proportion of T1 disease at explant at 81.3% (*p* = 0.007).

**Fig. 2:**
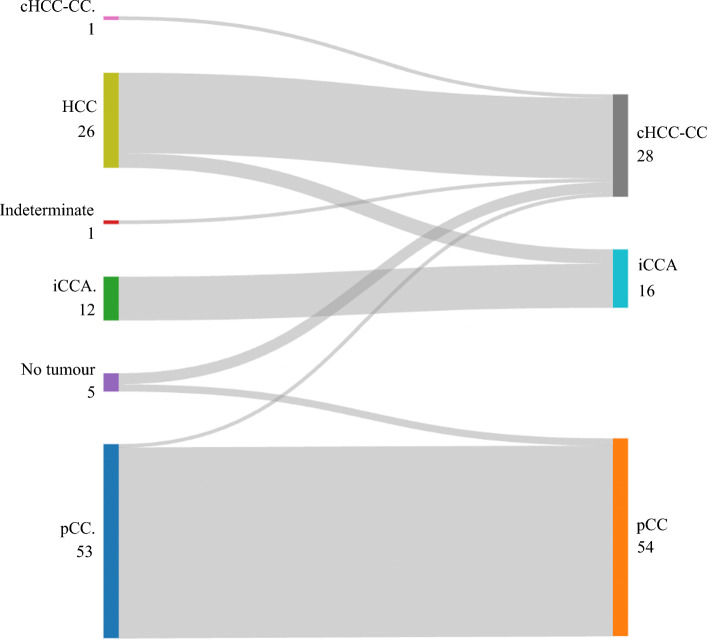
Preliver transplant tumor diagnosis with the final explant tumor diagnosis. *cHCC-CC* combined hepatocellular cholangiocarcinoma, *HCC* hepatocellular carcinoma, *pCC* perihilar cholangiocarcinoma, *iCCA* intrahepatic cholangiocarcinoma

Pretransplant diagnoses differed from explant pathology in 26 patients (26.5%) whereby 22/28 cHCC-CC (78.6%) were initially diagnosed as HCC, and 4/16 iCCA (25%) were also misclassified as HCC. By contrast, 53/54 pCC (98.1%) were correctly classified pretransplant (Fig. 2).

Overall, 89 patients (90.8%) received pretransplant antitumor therapy. Sixty-three (64.3%) received BTC-directed neoadjuvant therapy and 26 (26.5%) received HCC-directed locoregional therapy (predominantly TACE), reclassified as BTC only after explant pathology. For pCC, 52/54 (96.3%) received therapy, with 45 (83.3%) completing the institutional chemoradiation protocol and an additional 7 (13.0%) receiving SBRT, systemic chemotherapy, TACE, or combinations thereof. For iCCA, 14/16 (87.5%) received therapy including Y90 radioembolization (*n* = 8, 50.0%), systemic chemotherapy (*n* = 3, 18.8%), HAIP (*n* = 2, 12.5%), SBRT (*n* = 2, 12.5%), and ablation (*n* = 1). For cHCC-CC, 23/28 (82.1%) received locoregional therapy, predominantly TACE (*n* = 18, 64.3%), Y90 (*n* = 4, 14.3%) and ablation (*n* = 4, 14.3%), with 3 patients completing the pCC chemoradiation protocol owing to initial hilar cholangiocarcinoma diagnosis. The median waitlist time was 100 days [IQR 2–1290 days], which was shortest for pCC at 66 days (*p* = 0.022).

Among the 16 patients with iCCA, 11/13 with available radiologic data (84.6%) had solitary tumors and two (15.4%) were multifocal. Median pretreatment size was 4.8 cm [IQR 1.2–12.2 cm], decreasing to a median explant size of 1.2 cm [IQR 0–6.5 cm], demonstrating substantial treatment response. Three patients (18.8%) achieved complete pathologic response. Only two (12.5%) presented with a solitary tumor ≤ 3 cm on diagnostic imaging, meeting current UNOS exception criteria at diagnosis. After neoadjuvant therapy, seven (43.8%) had a solitary tumor ≤3 cm on pretransplant imaging. Among these seven patients, two (28.6%) recurred and one (14.3%) died, versus a 50% recurrence rate in the full iCCA cohort.

### Survival and Recurrence Outcomes

**Fig. 3:**
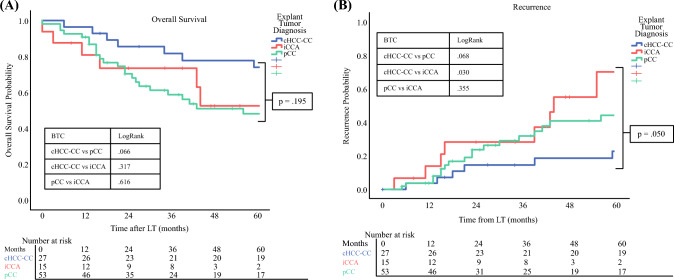
**A** Overall survival after liver transplantation by explant tumor diagnosis; **B** recurrence after liver transplantation by explant tumor diagnosis. *cHCC-CC* combined hepatocellular cholangiocarcinoma, *HCC* hepatocellular carcinoma, *pCC* perihilar cholangiocarcinoma, *iCCA* intrahepatic cholangiocarcinoma, *BTC* biliary tract cancer

The 1-, 3-, and 5-year OS for the cohort were 88%, 68%, and 58%, respectively. The mean interval from surgery to death was 37.7 months, with an average age at death of 64.3 years. The 1-, 3-, and 5-year OS for cHCC-CC were 97%, 82%, and 76%, respectively; for pCC they were 90%, 60%, and 49%, respectively; and for iCCA they were 81%, 73%, and 51%, respectively (Fig. 3A). Disease recurrence occurred in 40 patients (40.8%). iCCA had the highest recurrence rate at 50%, followed by pCC (42.6%) and cHCC-CC (32.1%, *p* = 0.472). The 1-, 3-, and 5-year recurrence probabilities were 9%, 27%, and 41%, respectively. Median time to recurrence was 16 months [IQR 1–160 months]. iCCA had the shortest interval (10 months, IQR 1–40 months), followed by pCC (15 months, IQR 4–155 months) and cHCC-CC (29 months, IQR 6–61 months, *p* = 0.462). The 1-, 3-, and 5-year recurrence probabilities for cHCC-CC were 6%, 15%, and 24%; for pCC they were 6%, 33%, and 45%, respectively; and for iCCA they were 17%, 29%, and 72%, respectively. iCCA showed significantly higher recurrence probability compared with cHCC-CC (*p* = 0.030) (Fig. 3B). Patients with recurrence had significantly higher mortality (87.5% versus 22.4%, *p* < 0.001). Patients with disease recurrence had a greater median post-neoadjuvant tumor size (2.5 cm) compared with those without recurrence (1.7 cm, *p* = 0.007) and a greater median explant tumor size (2.5 versus 2.0 cm, *p* = 0.029) (Table 2).

**Table 2:** Recurrence patterns, management, and survival outcomes based on explant tumor diagnosis

	cHCC-CC (*n* = 28)	pCC (*n* = 54)	iCCA (*n* = 16)	Total (*n* = 98)	*p*-Value
Time to recurrence (months), median [range]	29 [6–61]	15 [4–155]	10 [1–40]	16 [1–160]	0.462
Overall recurrence, *n* (%)	9 (32.1)	23 (42.6)	8 (50)	40 (40.8)	0.472
Early recurrence (≤12 months), *n* (%)	2 (22.2)	8 (34.8)	5 (62.5)	15 (37.5)	0.212
Late recurrence (>12 months), *n* (%)	7 (77.8)	15 (65.2)	3 (37.5)	25 (62.5)	
First recurrence location, *n* (%)					
Intrahepatic	1 (11.1)	5 (21.7)	2 (25)	8 (20)	0.736
Metastatic	8 (88.9)	18 (78.3)	6 (75)	32 (80)	
Oligo or metastatic disease, *n* (%)					0.567
Oligometastatic disease	8 (80)	17 (73.9)	4 (57.1)	29 (72.5)	
Metastatic disease	2 (20)	6 (26.1)	3 (42.9)	11 (27.5)	
Recurrence site, *n* (%)					
Lung	5 (55.6)	2 (8.7)	3 (37.5)	10 (25)	0.015
Intrahepatic	2 (18.2)	6 (26.1)	2 (28.6)	10	0.847
Distant lymph node	2 (22.2)	4 (17.4)	2 (25)	8 (20)	0.882
Distal small intestine	0	4 (17.4)	0	4 (10)	0.193
Pleura	2 (22.2)	0	1 (12.5)	3 (7.5)	0.084
Sigmoid colon	0	2 (8.7)	0	2 (5)	0.459
Other locations ^a^	1 (11.1)	11 (47.8)	2 (25)	14 (35)	0.118
Management of recurrence, *n* (%)					
Chemotherapy alone	5 (55.6)	12 (52.2)	4 (50.0)	21 (52.5)	0.614
Multimodal therapy	1 (11.1)	7 (30.4)	1 (12.5)	9 (22.5)	
Radiotherapy alone	1 (11.1)	0	1 (12.5)	2 (5.0)	
Supportive/palliative care	2 (22.2)	4 (17.4)	2 (25.0)	8 (20.0)	
Deaths, *n* (%)	13 (46.4)	29 (53.7)	6 (37.5)	48 (49.0)	0.497
Age at time of death, years (Mean ± SD)	69 ± 10	63 ± 10	61 ± 17	64.3 ± 11.2	0.202
Time from surgery to death, months (Mean ± SD)	50 ± 29	36 ± 38	20 ± 19	37.7 ± 34.2	0.192

^a^Vertebrae, pancreas, periumbilical skin, retroperitoneum, omentum, mediastinum, uterus, kidney, and adrenal gland

Early and late recurrence percentages are calculated among patients with recurrence

*cHCC-CC* combined hepatocellular cholangiocarcinoma, *HCC* hepatocellular carcinoma, *pCC* perihilar cholangiocarcinoma, *IHC* intrahepatic cholangiocarcinoma, *SD* standard deviation

### Recurrence Patterns by Subtype

Among 40 patients who developed recurrence, the most common sites were lung (*n* = 10) and liver (*n* = 10), followed by distant lymph nodes (*n* = 8) and distal small intestine (*n* = 4). The majority of recurrences were extrahepatic/metastatic (81%), with intrahepatic recurrence in 16% and combined intra- and extrahepatic disease in 3%. Overall median post-recurrence survival was 7.3 months (IQR 3.1–13.5 months).

Recurrence patterns revealed distinct subtype-specific differences in timing, anatomic distribution, and post-recurrence survival (Table 2). iCCA demonstrated predominantly early recurrence, with 5/8 (62.5%) occurring within 12 months, compared with 8/23 (34.8%) for pCC and 2/9 (22.2%) for cHCC-CC. cHCC-CC recurrences demonstrated marked lung tropism (5/9, 55.6%), whereas pCC recurrences were heterogeneous with liver (5/23, 21.7%), distant lymph nodes (4/23, 17.4%), and small intestine (4/23, 17.4%) as frequent sites. iCCA recurrences were distributed across lung (3/8, 37.5%), liver (2/8, 25.0%), and distant lymph nodes (2/8, 25.0%). Median post-recurrence survival was 10.6 months for cHCC-CC, 8.6 for pCC, and 5.1 for iCCA. Early versus late recurrence was not significantly associated with post-recurrence survival (log-rank *p* = 0.834).

Post-recurrence treatment varied by subtype. Chemotherapy was the most common modality, administered to 12 patients with pCC (52%), 4 patients with iCCA (50%), and 5 patients with cHCC-CC (56%) as single-agent therapy. Multimodal therapy (combining surgery, chemotherapy, and/or radiotherapy) was more frequent for pCC (7/23, 30%) than iCCA (1/8, 13%) or cHCC-CC (1/9, 11%). Supportive or palliative care alone was provided in four pCC (17%), two iCCA (25%), and two cHCC-CC (22%) cases. Patients receiving multimodal therapy had median post-recurrence survival of 16.1 months compared with 5.4 months for single-modality treatment. Surgical resection for isolated recurrence (*n* = 4, including Whipple, mesenteric resection, and lung wedge resection) yielded the longest post-recurrence survival (median 24.1 months), though treatment selection was inevitably influenced by patient fitness and disease burden.

### Predictors of Recurrence and Survival

Results of univariate Cox regression are presented in Table 3. On univariate analysis, lymph node positivity (HR 3.06, 95% CI 1.34–7.00, *p* = 0.008) and explant tumor size (HR 1.20 per cm, 95% CI 1.05–1.38, *p* = 0.006) were significantly associated with recurrence-free survival. Age, tumor subtype, T stage, CA 19-9, calculated MELD, histologic grade, waitlist time, cirrhosis, and neoadjuvant chemoradiation were not significant on univariate analysis for either OS or RFS.

**Table 3:** Cox proportional hazards regression for overall survival and recurrence-free survival

Variable	Univariate–overall survival		Univariate–recurrence-free survival		Multivariate–RFS	
	HR (95% CI)	*p*	HR (95% CI)	*p*	HR (95% CI)	*p*
Age (per year)	1.02 (1.00–1.05)	0.095	1.00 (0.98–1.03)	0.774	–	–
pCC (versus cHCC-CC)	1.58 (0.88–2.83)	0.123	1.24 (0.66–2.32)	0.503	1.27 (0.55–2.93)	0.570
iCCA (versus cHCC-CC)	1.03 (0.44–2.45)	0.940	1.83 (0.83–4.00)	0.131	2.94 (1.11–7.75)	0.030
T stage (T3/T4)	0.81 (0.29–2.25)	0.680	0.97 (0.34–2.74)	0.954	–	–
Lymph node positive	1.82 (0.77–4.34)	0.175	3.06 (1.34–7.00)	0.008	3.27 (1.32–8.10)	0.010
Tumor size (cm)	1.11 (0.97–1.27)	0.138	1.20 (1.05–1.38)	0.006	1.22 (1.06–1.40)	0.005
CA 19-9 elevated (> 37)ᵃ	1.63 (0.76–3.50)	0.208	1.18 (0.55–2.54)	0.677	–	–
Calculated MELD	0.97 (0.94–1.01)	0.117	0.97 (0.93–1.02)	0.216	–	–
Poorly differentiated	1.00 (0.47–2.14)	1.000	0.72 (0.30–1.72)	0.464	–	–
Waitlist time (months)	1.01 (0.97–1.05)	0.795	1.00 (0.95–1.06)	0.867	–	–
Cirrhosis	1.10 (0.53–2.28)	0.802	0.91 (0.42–1.97)	0.804	–	–
Neoadjuvant chemoRT	1.20 (0.68–2.15)	0.528	1.03 (0.55–1.93)	0.923	–	–

Multivariate model includes variables with *p* < 0.20 on univariate analysis

C-index: OS 0.607; RFS 0.676

ᵃCA 19-9 analysis on *n* = 70 with available data

*HR* hazard ratio, *CI* confidence interval, *RFS* recurrence-free survival, *OS* overall survival, *RFS* recurrence free survival

On multivariate Cox regression (Table 3), lymph node positivity (HR 3.27, 95% CI 1.32–8.10, *p* = 0.010), iCCA vs. cHCC-CC subtype (HR 2.94, 95% CI 1.11–7.75, *p* = 0.030), and explant tumor size (HR 1.22 per cm, 95% CI 1.06–1.40, *p* = 0.005) were independently associated with RFS (C-index 0.676). For OS, no variables reached independent significance, though tumor size (HR 1.16, *p* = 0.063) and lymph node positivity (HR 2.09, *p* = 0.140) showed directionally consistent trends. In a subgroup analysis of patients with available CA 19-9 data (*n* = 70), pretransplant CA 19-9 elevation (> 37 U/mL) was not independently associated with recurrence (HR 0.83, *p* = 0.681) or overall survival (HR 1.07, *p* = 0.877) on multivariate analysis.

### Subgroup and Waitlist Analysis

To address heterogeneity from incidentally diagnosed BTC, a subgroup analysis compared the 66 intentionally transplanted patients with 32 incidental cases (26 transplanted for presumed HCC, 5 with no pretransplant malignancy, and 1 with an indeterminate lesion). Recurrence rates were comparable (40.9% versus 40.6%, *p* = 1.000), as were OS (log-rank *p* = 0.490) and RFS (log-rank *p* = 0.475). On multivariate analysis limited to intentional pCC and iCCA (*n* = 53), tumor size remained significant (HR 1.17, *p* = 0.04), while lymph node positivity showed a consistent but nonsignificant trend (HR 2.48, *p* = 0.17), likely reflecting reduced statistical power. This sensitivity analysis confirms that the combined cohort findings are not driven by the incidental cases and supports the validity of analyzing all BTC subtypes together.

## Discussion

Existing data on LT for BTC remain limited and highly heterogeneous, with most evidence derived from small retrospective series and single-arm protocols.^6,10–13^ Current ASCO and ESMO guidelines prioritize systemic chemotherapy for advanced BTC, and recent consensus emphasizes significant uncertainty around optimal candidate selection, neoadjuvant strategies, and management of recurrence.^2,14^ Benchmark studies in pCC demonstrate that LT combined with standardized neoadjuvant therapy can outperform resection, yet these protocols are available at few centers.^15^ For iCCA, the international consortium by Sapisochin et al. reported 5-year survival of 65% for tumors ≤ 2 cm,^16^ and current UNOS policies allow exception consideration solely for solitary tumors ≤ 3 cm with 6 months of stability.^11^ Dageforde et al. reported 5-year OS of 70.1% for cHCC-CC within Milan criteria.^17^ Against this backdrop, this 18-year single-center series provides one of the largest prospectively characterized cohorts of BTC transplant recipients, offering granular data on subtype-specific biology, neoadjuvant exposure, recurrence patterns, and post-transplant outcomes that help fill critical gaps in the literature.^6,18^

The challenge in a multimodal era is to identify early those who are expected to derive a meaningful benefit from transplantation. Our center applies strict eligibility criteria in accordance with UNOS guidelines for LT for BTC. For unresectable pCC, many centers, including our own, have developed specific neoadjuvant chemoradiation protocols with significantly improved outcomes, offering long-term survival greater than 50%.^4,19,20^ This is in alignment with current UNOS exception point protocols for tumors measuring ≤ 3 cm and strategic preoperative staging to exclude patients with occult extrahepatic spread.^21^ The transplant community has adopted a more cautious approach for iCCA, and for many centers, this remains a contraindication for LT.^5^ There is no current consensus for larger tumors, and much of the evidence is limited to institutional experiences where a small number make it to transplant.^5^ Our institution considers all cases of unresectable iCCA with liver-only disease who have demonstrated a good response to neoadjuvant treatment and 6 months of disease stability for transplantation. Notably, while only 2 of 16 patients with iCCA (12.5%) met UNOS exception criteria at diagnosis (solitary tumor ≤ 3 cm on imaging), 7 (43.8%) achieved this threshold after neoadjuvant therapy, suggesting that treatment response may meaningfully expand the transplant-eligible population beyond what initial tumor size alone would predict.

Our 5-year OS of 58% is consistent with high-volume center reports.^6,18,22^ However, the appropriate comparator for unresectable BTC is the natural history of the disease, for which median survival with systemic chemotherapy alone remains approximately 11–12 months.^23^ Outcomes varied substantially by subtype: cHCC-CC achieved 76% 5-year OS, competitive with noncancer LT and HCC-transplanted cohorts, whereas pCC (49%) and iCCA (51%) had more modest results. The 40.8% recurrence rate underscores the need for refined patient selection, and our data suggest that molecular profiling and more granular size criteria, particularly the UNOS ≤ 3 cm solitary criterion for iCCA where eligible patients had only 28.6% recurrence, may help improve long-term outcomes but we want access to transplant for patients with unresectable larger tumors who may derive benefit.

The neoadjuvant landscape reflected cohort heterogeneity, with 64.3% receiving BTC-directed therapy and 26.5% receiving HCC-directed locoregional treatment for presumed HCC prior to reclassification on explant. The substantial treatment response observed in patients with iCCA, with median tumor size decreasing from 4.8 cm pretreatment to 1.2 cm at explant and an 18.8% complete pathologic response rate, supports the predictive value of radiologic response to aggressive neoadjuvant therapies in achieving satisfactory post-LT outcomes. Our results provide important data regarding the predictive ability of a good radiologic response to aggressive neoadjuvant therapies and may challenge some of the assumptions underpinning size thresholds for large iCCA.

Recurrence timing and site patterns differed substantially across subtypes. iCCA demonstrated predominantly early recurrence (62.5% within 12 months), consistent with aggressive biology, whereas cHCC-CC recurred late (78% at ≥ 12 months) with pulmonary tropism. pCC demonstrated more heterogeneous recurrence involving liver, lymph nodes, and small intestine. These patterns have implications for subtype-specific surveillance strategies and warrant investigation in larger cohorts. Management strategies for recurrence were individualized. Patients who underwent surgical resection for isolated recurrence demonstrated the longest survival, while multimodal therapy outperformed single-modality treatment. Contemporary surgical series similarly suggest that selected patients with oligometastatic or oligo-recurrent BTC can achieve favorable survival with aggressive locoregional treatment, such as repeat resection or radiotherapy, particularly when recurrence is limited in number and occurs late after initial surgery.^24,25^

On multivariate Cox regression, lymph node positivity, iCCA histology, and explant tumor size were independently associated with recurrence-free survival. Lymph node positivity conferred a more than three-fold increased hazard of recurrence (HR 3.27), underscoring the prognostic importance of thorough nodal assessment at transplantation. These findings are consistent with prior studies identifying tumor size and nodal metastases as key predictors of poor post-transplant outcomes in BTC.^7^ The finding that iCCA carried a nearly three-fold recurrence risk (HR 2.94 versus cHCC-CC) is consistent with emerging data suggesting more aggressive tumor biology in iCCA.^6,24^ Notably, pretransplant CA 19-9 elevation (> 37 U/mL) was not independently associated with recurrence or survival on multivariate analysis in this cohort. While CA 19-9 has been previously reported as a prognostic marker with higher levels correlating with increased recurrence risk,^18,21^ our results suggest that in the context of protocolized neoadjuvant treatment and strict selection criteria, its independent prognostic value may be attenuated. MELD score, histologic grade, and neoadjuvant chemoradiation protocol were also not significant predictors of recurrence or survival.

This study also highlights diagnostic discrepancies between pretransplant and explant classification, particularly in cHCC-CC and iCCA where imaging misclassified a significant proportion as HCC. The high rate of diagnostic discrepancy may partially reflect the evolution of imaging technology over the 18-year study period, as advances in cross-sectional imaging have improved the ability to characterize hepatic lesions and distinguish between tumor subtypes. This issue has been described in prior literature and emphasizes the need for enhanced diagnostic modalities, possibly involving molecular profiling pretransplant.^18,26–28^ While tumor subtype did not independently predict overall survival, iCCA was a significant independent predictor of recurrence, reinforcing the need for subtype-specific surveillance protocols following transplantation.

A subgroup analysis excluding the 32 incidental cases confirmed that the findings of the full cohort were robust. Recurrence rates (40.9% versus 40.6%, *p* = 1.000), overall survival (log-rank *p* = 0.490), and recurrence-free survival (log-rank *p* = 0.475) were comparable between intentional and incidental groups. This suggests that incidentally discovered BTC on explant pathology carries comparable oncologic risk, which is itself a clinically relevant observation.

Notably, waitlist attrition was almost exclusively among patients with pCC (18/19, 94.7%), with tumor progression or metastatic disease accounting for 15 of 19 removals (78.9%). Three patients were taken to the operating room but had transplantation aborted owing to metastatic disease discovered at exploration. This high dropout rate among pCC reflects the rigorous serial restaging inherent to the neoadjuvant chemoradiation protocol, which identifies disease progression early and prevents futile transplantation. These findings are consistent with Dong et al., who demonstrated significant waitlist dropout in LT for pCC compared with liver resection, underscoring the importance of intent-to-treat analyses when evaluating LT outcomes for BTC.^6^

Several limitations should be acknowledged. The single-center design and limited cohort size may restrict generalizability. Selection bias owing to referral patterns and protocol eligibility remains inherent. Precise terminology of treatment response and appropriate downstaging is increasingly required, especially for iCCA. Additionally, this cohort spans 18 years (2007–2025) and includes the institutional learning curve. Imaging protocols evolved over the study period in accordance with advances in cross-sectional imaging technology; this evolution may have influenced diagnostic accuracy across eras. The inclusion of three biologically distinct BTC subtypes introduces heterogeneity; however, subgroup analysis demonstrated comparable outcomes between intentional and incidental cases, and these subtypes share a common institutional pathway of multidisciplinary evaluation, neoadjuvant therapy, and transplantation.

## Conclusions

This analysis, the largest single-center BTC transplant series to our knowledge, supports LT as a promising and increasingly effective option for unresectable BTC when no other surgical option exists. Lymph node positivity, iCCA tumor subtype, and explant tumor size were identified as independent predictors of disease recurrence, providing a foundation for risk stratification in this population. Accordingly, we advocate for the enrollment of selected patients with unresectable BTC by specialist transplant centers with aggressive multimodal neoadjuvant therapy, thorough nodal assessment, and vigilant post-transplant surveillance. Future genomic insights may assist in the prognostication of survival and response to neoadjuvant protocols, which should be considered across international oncologic consortia.
